# Diagnostic value of duplex ultrasonography in comparison with conventional angiography in the assessment of traumatic arterial injuries of the extremities

**DOI:** 10.1186/cc9872

**Published:** 2011-03-11

**Authors:** H Ravari

**Affiliations:** 1 Imamreza, Mashhad, Iran

## Introduction

Traumatic events are one of the major causes of arterial injuries. Physical examination is not a good predictor of these injuries and arteriography is considered the gold standard for this purpose. In recent years, non-invasive modalities are increasingly replacing diagnostic arteriography. Duplex ultrasonography is an excellent method for investigation of arterial diseases. In this study, we analyze the diagnostic value of duplex ultrasonography in comparison with angiography in traumatic arterial injuries of the extremities.

## Methods

Duplex ultrasonography was performed for patients with suspicious arterial injury due to extremity trauma just before angiography. The Doppler pattern and flow states were obtained, then standard angiography was performed. The results of duplex ultrasonography were compared with angiography.

## Results

A total of 75 patients with blunt and penetrating trauma to their extremities were investigated. Duplex ultrasonography had 95% sensitivity and 98% specificity in the diagnosis of arterial injury in this study.

## Conclusions

We suggest that duplex ultrasonography can be used as a reliable tool, both sensitive and specific, in screening of hemo-dynamically stable patients with suspicious limb arterial injury.

